# Evaluation of Allogeneic Bone-Marrow-Derived and Umbilical Cord Blood-Derived Mesenchymal Stem Cells to Prevent the Development of Osteoarthritis in An Equine Model

**DOI:** 10.3390/ijms22052499

**Published:** 2021-03-02

**Authors:** Lélia Bertoni, Sandrine Jacquet-Guibon, Thomas Branly, Mélanie Desancé, Florence Legendre, Martine Melin, Pascaline Rivory, Daniel-Jean Hartmann, Amandine Schmutz, Jean-Marie Denoix, Magali Demoor, Fabrice Audigié, Philippe Galéra

**Affiliations:** 1 Center of Imaging and Research on Locomotor Affections on Equines (CIRALE), Unit Under Contract 957 Equine Biomechanics and Locomotor Disorders (USC 957 BPLC), French National Research Institute for Agriculture Food and Environment (INRAE), Ecole Nationale Vétérinaire dʹAlfort, F-94700 Maisons-Alfort, France; lelia.bertoni@vet-alfort.fr (L.B.); sandrine.jacquet@vet-alfort.fr (S.J.-G.); jean-marie.denoix@vet-alfort.fr (J.-M.D.); fabrice.audigie@vet-alfort.fr (F.A.); 2 BIOTARGEN, UNICAEN, Normandie Université, 14000 Caen, France; tbranly@gmail.com (T.B.); melanie359@hotmail.fr (M.D.); florence.legendre@unicaen.fr (F.L.); philippe.galera@unicaen.fr (P.G.); 3 NOVOTEC, ZAC du Chêne, Europarc, 69500 Bron, France; mmelin@novotec-labs.com (M.M.); privory@novotec-labs.com (P.R.); dhartmann@ novotec-labs.com (D.-J.H.); 4 CWD-VetLab, Unit Under Contract 957 Equine Biomechanics and Locomotor Disorders (USC 957 BPLC), French National Research Institute for Agriculture Food and Environment (INRAE), Ecole Nationale Vétérinaire dʹAlfort, F-94700 Maisons-Alfort, France; schmutz.amandine@gmail.com

**Keywords:** horse, mesenchymal stem cells, bone marrow, umbilical cord blood, allogeneic, osteoarthritis, pre-clinical study

## Abstract

Osteoarthritis (OA) is a significant cause of pain in both humans and horses with a high socio-economic impact. The horse is recognized as a pertinent model for human OA. In both species, regenerative therapy with allogeneic mesenchymal stem cells (MSCs) appears to be a promising treatment but, to date, no in vivo studies have attempted to compare the effects of different cell sources on the same individuals. The objective of this study is to evaluate the ability of a single blinded intra-articular injection of allogeneic bone-marrow (BM) derived MSCs and umbilical cord blood (UCB) derived MSC to limit the development of OA-associated pathological changes compared to placebo in a post-traumatic OA model applied to all four fetlock joints of eight horses. The effect of the tissue source (BM vs. UCB) is also assessed on the same individuals. Observations were carried out using clinical, radiographic, ultrasonographic, and magnetic resonance imaging methods as well as biochemical analysis of synovial fluid and postmortem microscopic and macroscopic evaluations of the joints until Week 12. A significant reduction in the progression of OA-associated changes measured with imaging techniques, especially radiography, was observed after injection of bone-marrow derived mesenchymal stem cells (BM-MSCs) compared to contralateral placebo injections. These results indicate that allogeneic BM-MSCs are a promising treatment for OA in horses and reinforce the importance of continuing research to validate these results and find innovative strategies that will optimize the therapeutic potential of these cells. However, they should be considered with caution given the low number of units per group.

## 1. Introduction

Degenerative joint disease also known as osteoarthritis (OA) is the most common joint disease found in both humans and horses with high socio-economic repercussions [1,2,3]. This disease is characterized by the degeneration of the articular cartilage, usually associated with damage to other components of the joint. Sclerosis of the subchondral bone, periarticular osteophytes, synovitis, and/or fibrosis of the periarticular tissues are frequent pathological findings associated to OA. All of these abnormalities are responsible for the clinical signs of osteoarthritis: stiffness, pain, and reduced range of joint motion [4]. In sport and race horses, the fetlock joint (metacarpo/tarso-phalangeal joint) is the most frequently affected [5]. In the racing industry, for example, injuries to the metacarpophalangeal joint have even been reported as the main cause of lameness, interruption of training and loss of race gains [6,7].

While some joint tissues such as bone have spontaneous healing properties, others such as articular cartilage do not: this highly specialized connective tissue is non-innervated and avascular and therefore has a very low repair capacity. As a result, traumatic and degenerative damage to articular cartilage inevitably progresses to OA and no treatment can currently stop this progression. In this context, cartilage regenerative medicine strategies based on intra-articular injection of stem cells represent a promising therapeutic tool thanks to their anti-inflammatory and pro-regenerative properties. Among the available sources of stem cells, adult mesenchymal stem cells (MSCs) are the most studied: they are easy to isolate from a multitude of tissues (bone marrow, adipose tissue, synovial membrane, muscle, periosteum, placenta, umbilical cord blood, peripheral blood, gums) and are not subjected to the ethical concerns of embryonic stem cells [8]. Allogeneic sources are of particular interest because banking can be done with ready-to-use characterized cells of known quality and quantity [4,9]. There is currently no consensus on the optimal source, dose, timing or number of stem cell injections that would be required to treat OA lesions in either horses [10] or humans [11,12]. Regarding the source, all MSC have the same functional qualities but many quantitative variations exist for their ease of isolation, their proliferative capacities, and their ability to differentiate into different cell lines. In our study, the choice was made for bone-marrow (BM) and umbilical cord blood (UCB), two tissue sources that have demonstrated to us the greatest benefit for targeted application. For example, it appears that MSCs derived from bone marrow (BM-MSCs) and umbilical cord blood (UCB-MSCs) have a greater ease of isolation, and a better proliferative capacity than MSCs derived from adipose tissue or peripheral blood [13,14,15,16,17]. In addition, both sources have an excellent chondrocyte differentiation potential [13,14], which has been shown to be better for BM-MSCs than for MSCs derived from adipose tissue or peripheral blood [15,18,19].

To date, few randomized controlled blinded equine studies have evaluated the effectiveness of intra-articular MSCs in the treatment of OA. Only one, published in 2009, has evaluated the effects of MSCs injected alone in carpal joints compared to a placebo in an OA model. This study compared two different cell products, namely autologous adipose-derived stromal vascular fractions and autologous BM-MSCs [20]. Three other controlled equine studies have evaluated the effects of MSCs in combination to a MSC vehicle (allogeneic equine plasma or autogenous fibrin) or in combination to a surgical technique [21,22,23]. To our knowledge, no equine study to date assesses the effectiveness of sole injections of allogeneic MSCs harvested from two different tissue sources, compared to a placebo on experimentally induced OA-lesions. 

The objective of this study is to evaluate the ability of a single intra-articular injection of allogeneic MSCs of controlled quality and viability, derived from two different tissue sources, namely BM-MSCs and UCB-MSCs, to limit the development of OA-associated pathological changes compared to placebo, in a post-traumatic osteoarthritis model applied to all four fetlock joints of eight horses. The hypothesis is that clinical, imaging, biochemical, macroscopic, and histologic OA-associated pathological changes develop less in MSC treated fetlocks than in placebo-treated fetlocks over a 12-week period. The secondary goal of this study is to compare treatment effects of BM-MSCs and UCB-MSCs with the hypothesis that BM-MSCs have a better ability to decrease the progression of OA than UCB-MSCs, according to a previous study indicating a better chondrogenic potential for BM-MSCs [24]. 

## 2. Results

### 2.1. Clinical Outcomes

**Figure 1:**
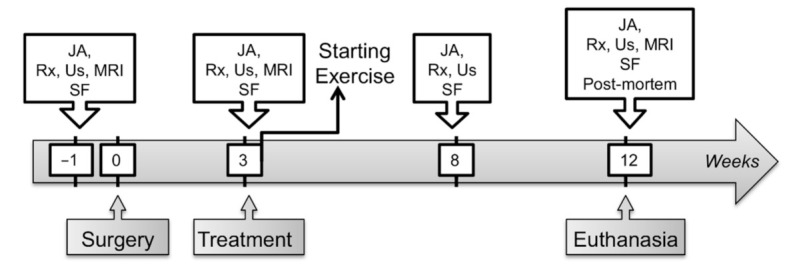
A schematic presentation of the timeline of the study. JA—Joint assessment; Rx—Radiographic examination; Us—Ultrasound examination; MRI—Magnetic resonance imaging; SF—Synovial fluid sampling and analysis; Post-mortem—Post-mortem analysis including macroscopic and microscopic examination of the joints after euthanasia.

**Figure 2:**
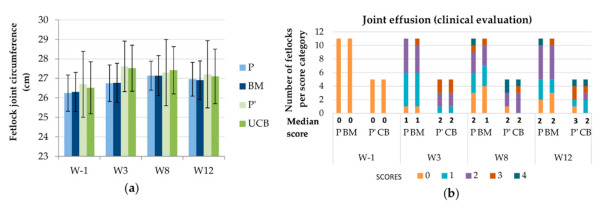
Evolution of two clinical parameters throughout the study period displayed per treatment group and per time point. (**a**) Mean (standard deviation) of the fetlock joint circumference. (**b**) Median scores and number of fetlocks per joint effusion score. W—Week; P—Fetlocks injected with placebo, contralateral of the fetlocks injected with bone-marrow derived mesenchymal stem cells (BM-MSCs); BM—Fetlocks injected with BM-MSCs; P’—Fetlocks injected with placebo, contralateral of the fetlocks injected with umbilical cord blood derived mesenchymal stem cells (UCB-MSCs); CB—Fetlocks injected with UCB-MSCs.

After the surgical procedure (Figure 1), no signs of discomfort were noted on clinical examinations and horses moved comfortably in their stall. No horse showed evidence of heat, pain or swelling at the surgery sites when bandage changes were performed. No adverse event was observed during the week following treatment (Dataset S1). None of the 8 horses showed sensitivity to digital flexion tests throughout the study period (Dataset S2). From inclusion one week before surgery (W-1) to the end of the study twelve weeks after surgery (W12), MSC treated fetlocks (either BM or UCB-MSCs) did not demonstrate significant differences in the evolution of fetlock joint circumference or fetlock joint effusion compared to placebo treated fetlocks (Figure 2).

### 2.2. Follow-Up with Imaging Techniques

**Figure 3:**
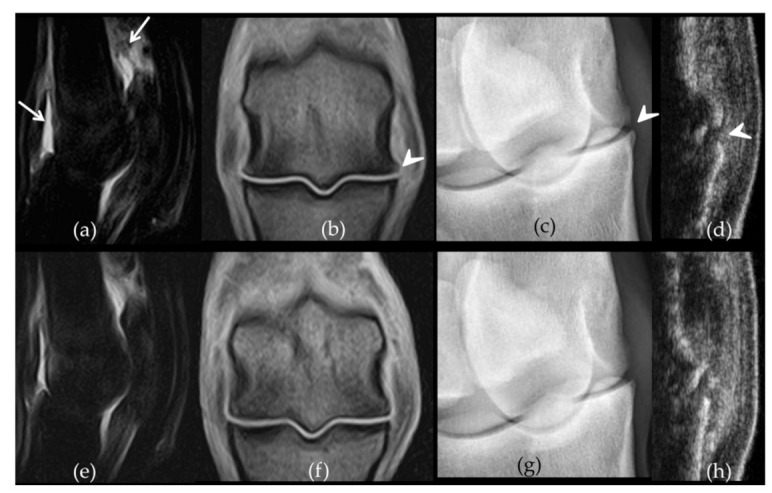
Comparative imaging findings observed on week 12 on the front metacarpophalangeal joints of horse 7. The left fore fetlock (**a**–**d**) received placebo treatment while the right fore fetlock (**e**–**h**) received bone-marrow derived mesenchymal stem cells (BM-MSCs) on week 3. On the left fetlock there was grade 3 (white arrows) synovial effusion (**a**) and periarticular osteophytes (**b**) (arrow heads) on Short Tau Inversion Recovery sagittal magnetic resonance imaging (MRI) images (**a**) and T1-weighed dorsal MRI images (**b**) respectively while grade 2 synovial effusion was observed on the right fetlock (**e**–**f**). Similarly, there are grade 3 (arrow head) osteophytes on the dorsomedial-palmarolateral 35° oblique radiographic view from the left fore fetlock (**c**) compared to a grade 1 on the right (**g**). The ultrasound osteophyte score was 3 on the left (**d**) (arrow head) and 2 on the right fetlock (**h**), on the longitudinal sections made on the dorso-lateral aspect of both joints.

**Figure 4:**
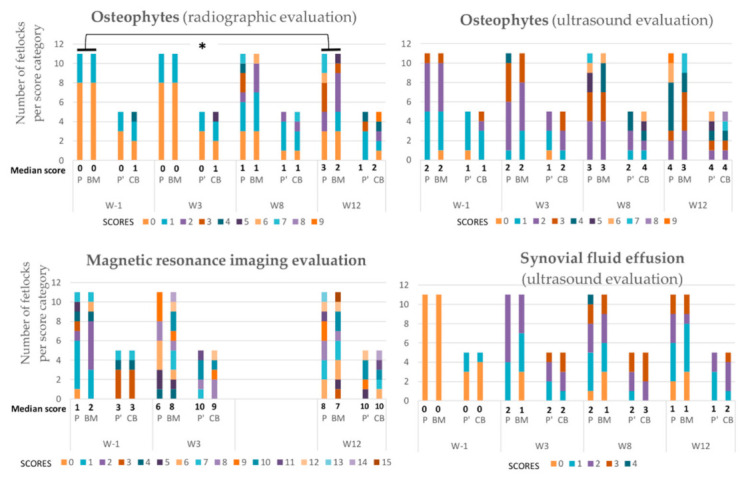
Evolution of the median scores and number of fetlocks per score category obtained for imaging parameters over the study period displayed per treatment group and per time point. W—Week; P—Fetlocks injected with placebo, contralateral of the fetlocks injected with bone-marrow derived mesenchymal stem cells (BM-MSCs); BM—Fetlocks injected with BM-MSCs; P’—Fetlocks injected with placebo, contralateral of the fetlocks injected with umbilical cord blood derived mesenchymal stem cells (UCB-MSCs); CB—Fetlocks injected with UCB-MSCs. * Significant difference between paired placebo and mesenchymal stem cell (MSC) treated fetlocks, considering the differences between W12 and W-1 values with *p* < 0.05.

**Figure 5:**
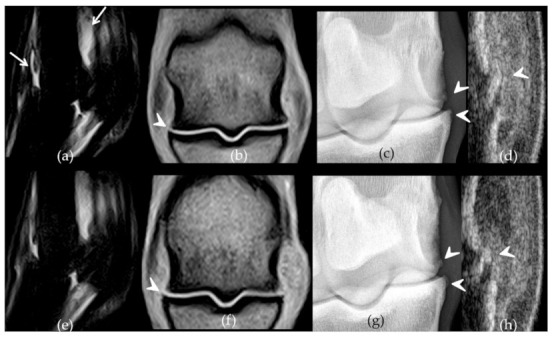
Comparative imaging findings observed on week 12 on the front metacarpophalangeal joints of horse 4. The left fore fetlock (**a**–**d**) received placebo treatment while the right fore fetlock (**e**–**h**) received umbilical cord blood derived mesenchymal stem cells (UCB-MSCs)on week 3. On the left fetlock there was grade 3 (white arrows) synovial effusion (**a**) and periarticular osteophytes (**b**) (arrow heads) on Short Tau Inversion Recovery sagittal magnetic resonance imaging (MRI) images (**a**) and T1-weighed dorsal MRI images (**b**) respectively, while grades 2 were observed on the right fetlock (**e**–**f**). There are grade 2 (arrow heads) osteophytes on the dorsomedial-palmarolateral 35° oblique radiographic views from both fore fetlocks (**c**,**g**) and on the dorso-lateral aspect of both joints on ultrasound (**d**,**h**) (arrow heads).

There was a significant difference between paired placebo and BM-MSC treated fetlocks in the development of OA-associated imaging signs (difference between W12 and W-1 values) with less severe imaging signs of OA developing in the BM-MSC treated fetlocks than in placebo treated fetlocks, when considering all four imaging parameters polled together (*p* = 0.006) (Figure 3). When considering imaging outcomes separately, there was also a trend towards a significant difference (*p* = 0.049) in the development of osteophytes on radiographs between BM-MSC treated fetlocks and placebo treated fetlocks (Figure 4). Indeed, osteophytes developed more from W-1 to W12 in placebo treated fetlocks (+2; 0.8–3.3) than in BM-MSC treated fetlocks (+1;0–2). No significant differences were detected between MSC sources and between paired placebo and UCB-MSCs fetlocks regarding imaging parameters (Figure 5).

### 2.3. Evolution of Synovial Fluid Parameters

**Figure 6:**
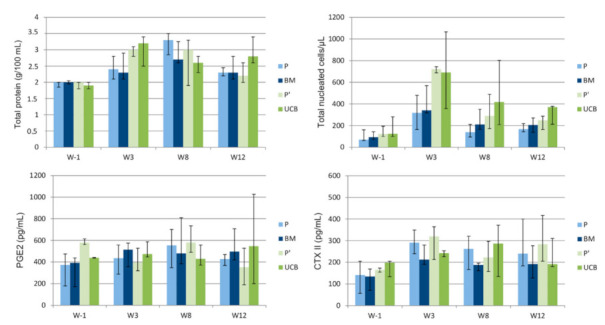
Evolution throughout the study period of the median values (1st quartile- 3rd quartile) of synovial fluid parameters displayed per treatment group. W—Week; P—Fetlocks injected with placebo, contralateral of the fetlocks injected with bone-marrow derived mesenchymal stem cells (BM-MSCs); BM—Fetlocks injected with BM-MSCs; P’—Fetlocks injected with placebo, contralateral of the fetlocks injected with umbilical cord blood derived mesenchymal stem cells (UCB-MSCs); UCB—Fetlocks injected with UCB-MSCs.

No significant differences in the evolution of synovial fluid total protein concentration and total nucleated cell counts were observed between treatments. Similarly, no significant treatment effects were seen on the concentration of synovial fluid biomarkers of inflammation and cartilage turnover (Figure 6).

### 2.4. Post-Mortem Evaluation

**Figure 7:**
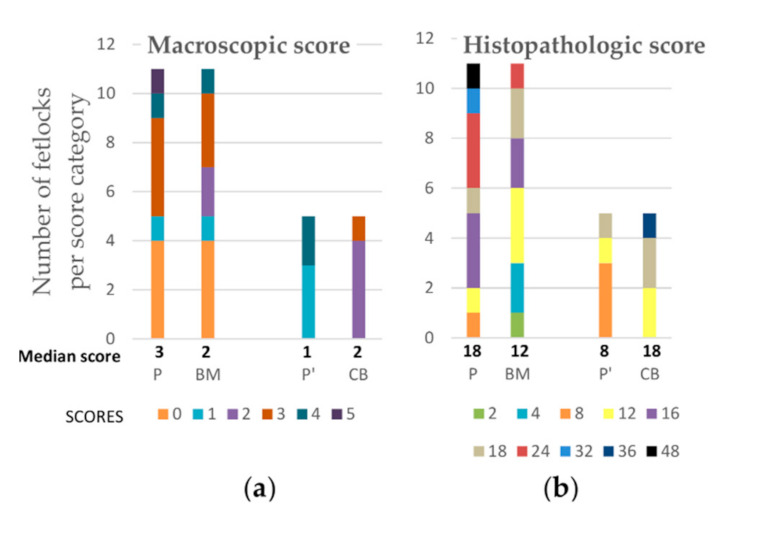
Median post-mortem scores and number of fetlocks per macroscopic (**a**) and histopathological (**b**) scores obtained on week 12 and displayed per treatment group. P—Fetlocks injected with placebo, contralateral of the fetlocks injected with bone-marrow derived mesenchymal stem cells (BM-MSCs); BM—Fetlocks injected with BM-MSCs; P’—Fetlocks injected with placebo, contralateral of the fetlocks injected with umbilical cord blood derived mesenchymal stem cells (UCB-MSCs); CB—Fetlocks injected with UCB-MSCs.

**Figure 8:**
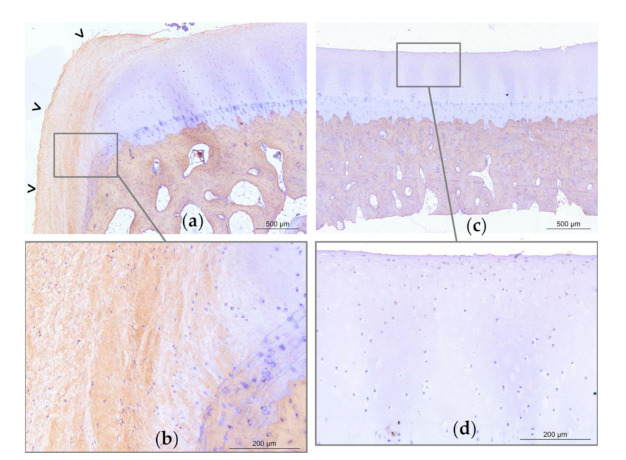
Representative light micrographs of osteochondral sections of the medial part of the distal aspect of the metacarpal condyles of horse 8. Micrographs obtained on Week 12, revealing type I collagen labelling (brown marking), and illustrating a grade 24 histological score on the left metacarpal condyle treated with placebo (**a**–**b**) and a grade 4 on the right metacarpal condyle treated with bone-marrow derived mesenchymal stem cells (BM-MSCs) (**c**–**d**). On the placebo treated joint (**a**), there is significant erosion of the superficial layer of the articular cartilage with a large fibrocellular tissue (open arrow heads) that is partly positive for type I collagen. On one extremity of the sample, cartilage and tidemark are no longer present (**a**). The subchondral bone appears trabecular. On the contralateral BM-MSC treated joint (**c**), the superficial layer of the cartilage is mildly eroded (**d**). There is no fibrocellular tissue, the tidemark is visible, and the subchondral bone is compact (**c**).

**Figure 9:**
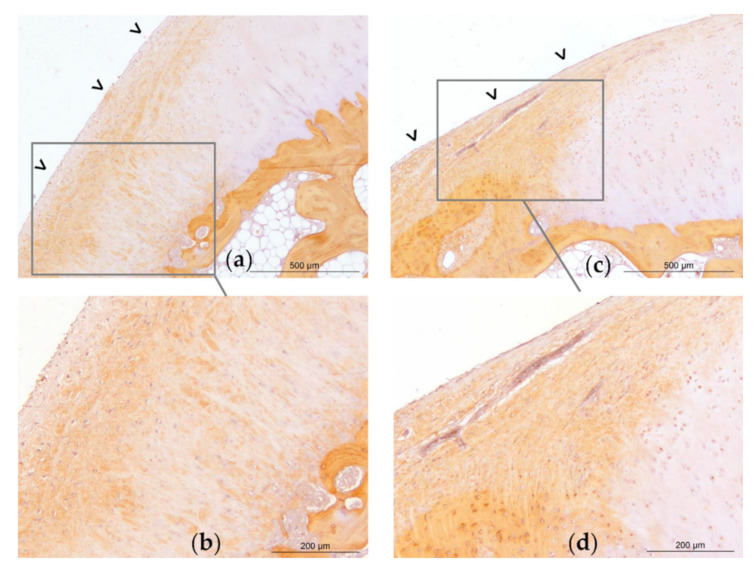
Representative light micrographs of osteochondral sections of the medial part of the distal aspect of the metacarpal condyles of horse 1. Micrographs obtained on Week 12, revealing type I collagen labelling (brown marking), and illustrating a grade 18 histological score on the left metacarpal condyle treated with umbilical cord blood derived mesenchymal stem cells (UCB-MSCs) (**a**–**b**) and on the right metacarpal condyle treated with placebo (**c**–**d**). On the UCB-MSC treated joint, there is mild erosion of the superficial layer of the articular cartilage (**a**). On both joints, there is a large fibrocellular tissue (open arrow heads) that is partly positive for type I collagen, and under which the tidemark is no longer present. The chondrocytes of the deep layers are also marked with type I collagen. The subchondral bone appears trabecular.

Gross examination at necropsy did not show significant treatment effects on OA-associated changes on metacarpal/tarsal condyles (Figure 7). Placebo treated fetlocks tended to have more severe histopathological scores (18;11–24) than BM-MSC treated fetlocks (12;8–17) (*p* = 0.07) (Figure 7 and Figure 8). There were no significant differences in histopathological scores between MSC sources and between UCB-MSC and placebo treated fetlocks (Figure 7 and Figure 9).

## 3. Discussion

The current study used a model of OA that was proven to successfully induce mild osteoarthritis-associated changes in the four fetlock joints of each horse [25]. OA-associated pathological changes in this model included clinical, radiographic, ultrasonographic, and magnetic resonance imaging (MRI) signs as well as biochemical modifications of synovial fluid composition and postmortem microscopic and macroscopic lesions. In the present study, 2 allogeneic MSC types from two different tissue sources, previously characterized and controlled for their quality and viability [13,14], were evaluated. The main finding of the present study is a significant reduction in the progression of OA-associated changes observed with imaging techniques for allogeneic BM-MSC treated fetlocks compared to their contralateral placebo treated fetlocks, when all imaging data was considered together. In particular the radiographic signs of OA were significantly lower for BM-MSC treated fetlocks compared to the placebos when considered alone. This difference with placebo treated fetlocks was not observed when injecting UCB-MSCs. The expected effects of MSCs in the treatment of diffuse OA lesions are mainly based on their paracrine activity, with the release of anti-inflammatory and pro-regenerative factors from extra-cellular vesicles [26]. MSCs from different tissue sources have many similarities, but exhibit differences in their protein expression profiles [27]. For example, a proteome analysis comparing BM-MSCs and UCB-MSCs in pigs revealed a high number of differentially expressed proteins involved in various aspects of cell biology, especially cell motility, with porcine BM-MSCs showing a higher migration capability than UCB-MSCs [28]. Similarly, it has been shown in vitro that during the process of chondrogenic differentiation, both equine MSC types expressed typical hyaline cartilage-associated molecules (like type II collagen) and other undesirable molecules (like type I collagen). However quantitative differences were measured between both cell sources, with a more favourable ratio toward hyaline cartilage-associated molecules for BM-MSCs [24]. To date, the content of extra-cellular vesicles of equine BM and UCB-MSCs has not been characterized, but it can be hypothesized, in order to explain the results of our study, that once injected in an inflammatory joint, BM-MSCs secrete more anti-inflammatory and pro-regenerative components than UCB-MSCs. Therefore, the synovial fluid biomarkers used in our study may not have been the most relevant, and other biomarkers should be considered in future studies.

These results suggest that intra-articular injection of a 10 million dose of allogeneic BM-MSCs into fetlock joints, 3 weeks after surgical induction of OA, has a beneficial effect on limiting the progression of imaging signs of OA. To our knowledge, this is the first time that a difference in the monitored imaging parameters has been demonstrated between placebo and treated joints during a short-term study (12 weeks) in a large animal model of OA. The results of the present study must however be examined with caution, considering the low number of units per group, especially for the group which received the UCB-MSCs, but also considering the absence of correction for multiple testing. In addition, only short-term effects (9 weeks) of these therapeutic MSCs were assessed. 

A lower number of treated joints per group had already shown significant results in a previous study evaluating the efficacy of blood derived chondrogenically induced stem cells combined to allogeneic equine plasma, for which there was also no correction for multiple testing or repeated measures [22]. In this previous study consisting in injection of a dose of 2 million cells, 5 weeks after the creation of the surgical lesions, significant improvements in the degree of lameness and synovial effusion score were observed compared to placebo, with also less severe macroscopic and microscopic scores, 11 weeks after the induction of the lesions. Compared to that study, the clinical and postmortem results of the present study are overall less conclusive. This could be explained by several variants in the experimental protocols. Firstly, the experimental design of our study, by creating simultaneous lesions on the 4 fetlocks of the same horse, did not make it possible to evaluate the degree of lameness of each limb. This is one of the limitations of our study, especially since lameness is one of the main symptoms of OA. Moreover, it is one of the most frequently improved symptoms in experimental equine studies of the effectiveness of OA treatments [29,30,31]. Secondly, another limitation of the present study is the potential systemic effect of MSCs that could have improved placebo treated joints, thus reducing the differences between groups. This effect was considered as insignificant in this study because systemic effects of MSCs have only been proven after their systemic administration [32]. Moreover, it has been reported that the large size of MSCs promotes passive cell entrapment and reduces their trafficking abilities [33]. The synovial membrane, surrounding the joint space constitutes a real blood to joint barrier that might particularly limit MSC trafficking. Nonetheless, small extracellular vesicles secreted by MSCs and responsible for their paracrine effects could have gained the systemic circulation [26]. Finally, although the study durations were rather similar (11 vs. 12 weeks), the model of surgical induction of lesions, combining osteochondral fragmentation of the proximal phalanx with chondral lesion generation [22] was therefore more severe than the osteochondral fragmentation alone.

The first published randomized blinded controlled experimental equine study evaluating effects of MSCs in the treatment of OA [20] had failed to demonstrate a beneficial effect of the injection of 5–15 million autologous BM-MSCs, 2 weeks after surgical induction of carpal OA. This 10 week duration study included clinical and radiographic evaluation, as well as synovial fluid analysis and macroscopic and microscopic post-mortem analysis. Similarly, another study evaluating injection of 12 million autologous BM-MSCs combined to a fibrin vehicle in an equine OA model did not demonstrate long-term (8 months) microscopic improvement of full thickness cartilage defects. In these studies, the cells were autologous, implying that there could be interindividual qualitative variations in the injected cells. The use of allogeneic products allows a standardization of the injected MSCs and reduces individual variations that could negatively impact the results. 

Our study has the advantage of using quality-controlled cells, but other factors related to the injection should be considered in order to improve the efficacy of the MSC-based therapies evaluated in this study. First, the dosage of injected cells might be lowered. Indeed, significant effects were obtained in the study of Broeckx et al. (2019) [34] when using 2 million cells. Similar results have been obtained in human clinical trials in which better improvements in pain levels and function were obtained on knee OA when using lower doses of MSCs [35,36]. Most published clinical studies with favorable results in horses generally use doses between 10 and 50 million cells [10], which is why the dose of 10 million was chosen in the present study. Repeated injections could also be considered in future studies. Indeed, even though most of the efficacy studies on MSCs have focused on evaluating the effectiveness of a single injection, there is growing evidence that repeated injections might be beneficial. Two recent controlled studies, one in rats [37] and the other in humans [38], have for instance demonstrated better clinical results with repeated injections, because it supposedly leads to a longer exposure of the joint to MSCs and therefore to their metabolic activity [37]. Second, the timing of injection could be reviewed. Outcome of clinical studies in horses evaluating MSCs seem to indicate that better results are obtained when administering MSCs past the inflammatory phase of injury [10,39]. It has indeed been demonstrated in vitro that the paracrine activity of MSCs varied depending on the environment in which they were placed. In particular, in an inflammatory environment (presence of lipopolysaccharide or Interleukine-1β), the production of pro-inflammatory cytokines (Tumor Necrosis Factor-α, Interleukine-6, Interleukine-8, Interleukine-1β) increases [40,41]. In future studies, it might therefore be beneficial to further delay the treatment from surgery. Third, the transport medium in which the cells are placed could also play a role in maintaining cell viability and optimizing its metabolic activity once located in the joint space. The use of Platelet Rich Plasma as a vehicle for MSCs has thus been used in several clinical equine studies with favorable results on the lameness score [34,42,43]. Efficacy of platelet concentrates in promoting wound healing and tissue regeneration has been at the center of scientific debate over the past few decades [44]. However, the benefit of this association compared to the injection of MSCs alone has so far only really been demonstrated in small animal models [45,46]. Very favorable results were also obtained in a goat model when autologous BM-MSCs were injected in combination with hyaluronic acid versus hyaluronic acid alone as a control. According to a recent review on injectable systems for intra-articular delivery of MSCs for cartilage treatment [47], it appears that 3 experimental studies in small animal models have demonstrated a beneficial effect of that combination compared to the injection of MSCs alone [48,49,50]. On another note of this review [47], the interest of hydrogels as stem cell vectors was also highlighted with the use of MSCs encapsulated in a self-assembling peptide hydrogel, namely KLD-12, a 12-residue peptide alternating hydrophilic and hydrophobic side groups, allowing self-assembly into ordered nanostructures that also present tissue-engineering properties [51]. A study in rats has indeed reported a beneficial effect of the injection of MSCs encapsulated in KLD-12 self-assembling peptide hydrogels compared to the injection of MSCs alone [52]. There is growing evidence that MSCs would gain therapeutic interest when combined with extracellular matrix substitutes in order to enhance their viability and metabolic activity, which suggests that future clinical applications of stem cell therapies will not aim to inject MSCs alone or simply diluted in Phosphate-Buffered Saline (PBS) [12,53]. Finally, induction of MSCs toward the chondrogenic lineage prior to their implantation, as performed in published equine experimental and clinical controlled studies [22,34], could also be a strategy to improve their therapeutic efficacy in OA.

Both MSC sources consisting of BM-MSCs and UCB-MSCs injected in the present study had already been evaluated in a previous safety study [54]. In that safety study, MSC injections induced mild to moderate local inflammatory signs compared to the placebo with individual variability in the inflammatory response. In the present study, MSC injections were performed with a simultaneous non-steroidal anti-inflammatory drug (NSAID) intravenous injection and no adverse events were observed. This indicates that NSAID injection might be effective to prevent local inflammatory reactions frequently associated with MSC injections [55,56,57] and could be recommended in practice as previously reported [57,58].

## 4. Materials and Methods

### 4.1. Horses and Study Design

Eight clinically sound French Standardbred horses owned by the Center of Imaging and Research on the Equine Locomotor Affections (CIRALE, Goustranville, France) were included in this blinded, controlled, randomized study. There were 4 geldings and 4 mares, median age was 3 years (ranging from 3 to 4), and median weight was 440.5 kg (ranging from 402 to 483 kg). None of the horses had a history of pregnancy, had received a blood transfusion before recruitment, or had kin relationships with MSCs donors. All horses had been trained for racing before being retired because of failure to trot at racing speeds. All were treadmill conditioned before the beginning of the study. 

The eight horses were divided into two groups. The first was composed of five horses whose fetlocks were assigned to one of three treatments groups: (1) allogeneic BM-MSCs, (2) allogeneic UCB-MSCs or (3) placebo control. The second was composed of three horses whose fetlocks were assigned to only two treatments groups: (1) BM-MSCs or (2) placebo control, in order to obtain more data on the cell source expected to have more favorable results according to the literature [18,24], and thus increase the statistical power for the evaluation of this treatment.

All horses received a single intra-articular treatment in all 4 fetlock joints, i.e., in both metacarpo-phalangeal and both metatarso-phalangeal joints, 3 weeks after surgical induction of OA lesions (W3) (Figure 1). An independent operator performed all injections so that evaluators were unaware of treatment assignments. In each of the eight horses included in the study, one fetlock of each pair of fore or hind limbs was injected with MSCs while the contralateral fetlock joint was injected with the same volume of placebo, consisting in the MSCs transport medium (Gibco PBS, Fisher Scientific SAS, Illkirch, France). Fetlocks were randomly selected to ensure that an equal number of right and left fetlocks were injected with stem cell-based therapy. For the first group of 5 horses whose fetlocks were assigned to one of three treatment groups, random selection assured an equal distribution of the two stem cell sources between the fore and hind fetlocks. Each horse had then one fore/hind fetlock joint injected with BM-MSCs and one front/hind fetlock joint injected with UCB-MSCs.

All procedures described in this study were approved by the ComEth Anses/ENVA/UPEC Ethical Committee (Date of approval: 10 March 2015, Permit number: 10/03/15-12). Surgeries were performed under general anesthesia and all efforts were made to minimize suffering and stress.

### 4.2. Inclusion and Induction of Osteoarthritis

One week before surgical induction of the lesions (W-1), each horse was evaluated clinically for lameness and underwent full radiographic and ultrasonographic examination of its four fetlock joints to rule out the presence of pre-existing OA before being included in the study (Figure 1). Induction of OA was performed on week 0 (W0) as previously described [25] by creating an osteochondral chip fragment in both metacarpophalangeal and metatarsophalangeal joints of each horse. Briefly, the dorsal edge of the fragment was freed from the proximal phalanx and the fractured bed was exposed and debrided to form a 15 mm wide defect bed. Debris from this procedure was not actively flushed in order to maximize joint inflammation. Operated joints were then routinely closed and bandaged, and postoperative care was performed following routine clinical standards. Horses were stall rested from surgery until the day after W3 injections, to be then turned out in small paddocks (10 × 10 m) until the end of the study. To enhance the induction of OA, exercise was initiated progressively on W3 (Figure 1) and consisted of 3 days per week work on a high speed treadmill for a 2 minutes trot (5 m/s), 2 minutes high speed trot (9 m/s) and 2 minutes trot (5 m/s) similarly as reported [29,59]. Days of treadmill exercise were alternated with 2 days of lunged trotting for 25 minutes equally distributed between right and left circles. 

### 4.3. MSC Isolation, Culture, Characterization, and Preparation

All MSC lines used in the present study were cryopreserved MSCs that had been previously isolated, cultured and characterized for the needs of a previously published safety study [54]. Briefly, after isolation, cell expansion was performed in low-glucose Dulbecco’s modified Eagle Medium containing 20% fetal calf serum (FCS, Invitrogen Life Technologies, Carlsbad, CA, USA). MSCs from passage 3 (P3) to passage 4 (P4) were immunophenotyped for expression levels of Major Histocompatibility Complex (MHC) class II and a panel of markers using flow cytometry. Cluster of Differentiation (CD) CD29, CD44 and CD90 expression were detected for all cell populations of each strain, and CD45 and MHC class II expression were not detected. CD73 expression was donor-dependent, with expression by only part of the cell population. Similarly, CD105 was not detected for UCB-MSCs and was weakly expressed by BM-MSCs at P4, as shown in other published reports [13,14]. The capacity of equine MSCs to differentiate into osteogenic, chondrogenic, and adipogenic lineages was determined at P4. All MSC strains used in the present study had high proliferative capacity and possessed multipotent capacity to differentiate into osteoblasts, adipocytes and chondrocytes, as previously described [13] except for UCB-MSCs that had only partial mesenchymal lineage differentiation ability, with no adipogenic differentiation obtained [14]. MSC lines were tested for nine viral genera, eight bacterial genera, and two protozoa by an external laboratory according to their internal protocols (Labéo Frank Duncombe, Saint-Contest, France) to ensure their safety. Passage 4 cells, kept per cell lines, were counted, centrifuged, suspended in a cryopreserved medium (6–10 million cells/mL) composed of 90% FCS and 10% dimethyl-sulfoxide (Sigma-Aldrich, Saint-Louis, MO, USA) and stored in liquid nitrogen. 

Two lines of BM-MSCs and 2 lines of UCB-MSCs from the safety study [54] were randomly assigned to the fetlocks of the horses of the present study. Approximately one week before the intra-articular injections, cells from selected cell lines were reseeded at 5000 cells/cm² and cultured with the cell expansion medium described above. Two to three hours before injection, cells were resuspended in 50 mL of PBS to completely remove the culture medium, and especially the residual FCS. Cells were then counted before a second centrifugation. Batches of MSCs containing 5 million cells/mL of PBS were prepared for injections. MSCs were maintained at room temperature (19–22 °C) during transport from the laboratory, as recommended [60]. Cell viability at the time of injection was controlled in the present study in the same conditions as the previous safety study [54].

### 4.4. Treatment

All horses were treated on W3 (Figure 1). Placebo treated fetlocks received 2 mL PBS. Fetlocks injected with MSCs received 10 million cells contained in 2 mL PBS. All injections were performed by a same trained operator who was not further involved in this study. Injections were done under sedation (intravenous administration of a combination of detomidine 0.01 mg/kg and butorphanol 0.01 mg/kg) after aseptic preparation of the skin. A lateral approach on the flexed limb was used with a 20-gauge needle, as recommended to ensure MSCs viability [60], inserted between the metacarpal/metatarsal condyle and the lateral proximal sesamoid bone. To avoid any joint flare reaction after intra-articular injections of MSCs, all horses received a concomitant intravenous injection of NSAID with 1.1 mg/kg flunixin meglumine (Finadyne, Intervet, Anger, France).

### 4.5. Clinical Assessment of the Joint, Investigation with Imaging Techniques, and Synovial Fluid Analysis

The protocol used was the same as the one previously described for the characterization of OA-associated changes in placebo treated fetlocks [25]. To control the safety of the procedure, clinical examinations (with measures of the vital parameters) were performed twice daily for one month following surgery, to check for signs of discomfort. Lameness examinations (including physical and dynamic examinations) were also performed on W3 (before treatment injection) and then 1, 3, and 7 days after injection to check any treatment adverse effect (i.e., a sudden increase in clinical grades ≥2) leading to a change in the weight bearing of the limbs that could impact the results of the present study. Clinical, radiographic, ultrasonographic, and MRI evaluation as well as synovial fluid sampling of each fetlock joint were performed on W-1, W3, W8, and W12 except for MRI that was not performed on W8 (Figure 1). Clinical evaluations were performed by 2 equine locomotor pathology specialists (SJ and LB). Images from radiography, ultrasonography and MRI were retrospectively evaluated blindly by 2 board-associated veterinary imaging specialists (FA and JMD). Clinical and imaging scores were fixed by consensus agreement. Parameters with different scores assigned by the observers were reviewed again and scores were discussed until a final score could be assigned by consensus.

Clinical evaluations consisted in fetlock joints circumference measurements, sensitivity to static digital flexion tests (grade 0–4), and joint effusion (grade 0–4) (Table S1). Degree of lameness was graded on a scale of 0 to 5 in accordance with the lameness scale of the American Association of Equine Practitioners [61]. As bilateral lameness could not be evaluated and graded given the study design, lameness data was monitored for complementary information but was excluded from statistical analysis. 

Images from radiographic and ultrasonographic evaluation of the fetlock joints were evaluated for osteophyte formation on the dorsal, lateral and medial aspects of the joint and scored based on osteophyte size (0 = none, 1 = small, 2 = medium, 3 = large) as previously reported [62,63]. The degree of synovial fluid effusion was also graded on ultrasound images with the same 5 points scale as for clinical evaluation (Table S2). Magnetic resonance images obtained on the horse under standing sedation using low field strength 0.27 Tesla Magnet (Hallmarq Veterinary Imaging, Guildford, Surrey, UK) (Table S3) were graded using a previously described semi-quantitative scoring system [64] adapted for the use in equine fetlock joint standing MRI. Both proximal phalanx and metacarpal/tarsal condyle were assessed for the presence of bone marrow oedema-like lesions (0–3), subchondral bone sclerosis (0–3) and osteophyte formation (0–3). Soft tissue structures associated to the metacarpo/tarsophalangeal joints were also graded for synovial fluid effusion (0–3), synovial membrane thickening (0–3), joint capsule oedema (0–3), and joint capsule thickening (0–3). A maximum cumulative score of 27 was possible (Table S4). 

Synovial fluid samples were assessed for total protein concentration using a refractometer and for total nucleated cell counts using an automatic analysis system (Sysmex XN10, Sysmex Corporation, Kobe, Japan) (cells/µL). The remaining fluid was stored at −80 °C for ELISA analysis. Synovial fluid concentration of Prostaglandin E_2_ (PGE_2_) and C-terminal of type II collagen (CTX-II) as markers of inflammation and cartilage turnover respectively, were estimated by use of commercially available high-sensitivity enzyme immunoassay kits (Prostaglandin E_2_ Parameter Assay Kit, R&D systems, Minneapolis, MN, USA; Serum Pre-Clinical CartiLaps^®^ (CTX-II) EIA, Immunodiagnostic Systems Holdings PLC, Boldon Business Park, UK) [65,66].

### 4.6. Postmortem Examination

Horses were subjected to euthanasia on W12 (Figure 1) with an intravenous injection of a mixture of 1g embutramide, 2.5 g mebezonium and 250 mg tetracaine hydrochloride (T-61, Intervet, Angers, France). Both metacarpophalangeal and both metatarsophalangeal joints of each horse were specifically examined and photographed. OA-associated changes on metacarpal/tarsal condyles were blindly scored according to the Osteoarthritis Research Society International (OARSI) Guidelines [67] by 2 investigators (LB and SJ) for wear lines (grade 0–3), erosions (grade 0–3), and osteochondral lesions (grade 0–3) (Table S5).

Osteochondral sections were harvested from the medial part of the distal aspect of the metacarpal/tarsal condyle and processed as previously described [25] for histological and immunohistological analysis. After fixation with neutral buffered formalin (Carlo Erba Reagents, Val-de-Reuil, France) and setting in paraffin, latero-medial slices 5 mm thick from the medial part of the distal aspect of the condyle were made. Sections were incubated overnight at 4 °C with rabbit anti-human type I collagen primary antibody (Novotec, 20111, Bron, France) or rabbit anti-human type II collagen (Novotec, 20211, Bron, France). After inhibition of the endogenous peroxidases by hydrogen peroxide, the sections were incubated in a peroxidase-conjugated secondary antibody (Dako, Envision rabbit, ref. K4002, Agilent Technologies, Santa Clara, CA, USA). The reaction with diaminobenzidine substrate (Dako, K3468, Agilent Technologies, Santa Clara, CA, USA) reveals the antigen-antibody complexes through the appearance of brown staining. The sections were counter-stained with Mayer hematoxylin then mounted between the slide and coverslip in an aqueous medium. The primary antibody was replaced by 3% PBS-diaminobenzidine as a negative control. Lesions were blindly scored according to the OARSI histopathology evaluation system [68] by 2 independent investigators (MM and DJH) for the severity of cartilage and bone lesions (grade 0–6), proportion of affected area by total area (grade 0–4), and lesion depth (grade 0–4). The total histological score of the lesions is the multiplication of those 3 parameters (Table S5).

### 4.7. Statistical Analysis

Development of OA-associated changes was compared between BM-MSC and placebo treated fetlocks pairs, and between UCB-MSC and placebo treated fetlock pairs, respectively. An overall evaluation of changes from the pre-osteoarthritis state to the post-osteoarthritis state (surgery combined to exercise) was made by comparing values resulting from the difference between W12 and W-1 values. An exception was made for the post-mortem scores that were analyzed on W12 only. For the imaging parameters, the 4 outcomes were analyzed separately but also as a group where all imaging scores were polled together by treatment category and analyzed as an additional outcome. Data was visually assessed for normality, and two different tests depending on the distribution of the variables were used. Differences in fetlock joint circumference, total nucleated cell counts and PGE_2_ concentration were normally distributed. When the outcome was not normally distributed, the non-parametric Wilcoxon signed-rank test was performed while paired Student’s *t*-test were used when the outcome was normally distributed. In addition, in order to compare treatment effects between the 3 treatment groups, values resulting from the difference between W12 and W-1 were analyzed using a one-way repeated measure analysis of variance (ANOVA) with the horse as a random effect for normally distributed outcomes, or a Kruskal-Wallis test for the others. The variables were finally presented descriptively, stratified by study date and treatment group. The mean and standard deviation of normally distributed outcomes and the median and quartiles of the others were calculated at each time point for each treatment groups. All statistical analyses were performed using R software (version 3.4.3; R Foundation for Statistical Computing, Vienna, Austria) or Excel 2016 for Windows (Microsoft, Redmond, DC, USA). A *p*-value of 0.05 was considered to be significant.

In the absence of available data on the efficacy of the studied cell-based therapies, an exact sample size could not be determined. Nevertheless, based on previous studies using MSCs [22] a MSC treatment success of 80% was expected and a placebo effect of improvement of approximately 20% was expected. Five fetlocks per treatment group results in 70% chance of detecting a difference between treatment groups, as significant at the 5% level. By increasing the number of BM-MSCs and placebo treated fetlocks to 11 (using 3 more horses), the chance to detect a difference between BM-MSCs and placebo treatments increases up to 95%.

## 5. Conclusions

To conclude, in an inflammatory environment, intra-articular injection of a 10 million dose of allogeneic BM-MSCs into fetlock joints of experimental horses, 3 weeks after surgical induction of OA, significantly reduces the progression of imaging signs of OA compared to contralateral placebo treated fetlocks, in particular regarding the formation of osteophytes on radiographs. No significant differences in the development of OA-associated pathological changes were observed between UCB-MSC treated fetlocks and their contralateral placebo treated joints. Therefore, allogeneic BM-MSCs could potentially be a promising treatment of OA in horses. These results are however to be tempered given the small number of horses and the lack of correction for multiple testing. In addition, the experimental design of this study does not rule out a potential systemic effect of the MSCs on placebo treated joints. As a step toward improvement of the efficacy of stem-cell based therapies in the treatment of OA in the future, either used alone or combined to biomaterials as vehicles, or even through injection of MSC extra-cellular vesicles, this study highlights the need to pursue investigation on the best modalities of administration and on strategies to potentiate their therapeutic effects.

## Data Availability

The data presented in this study are available in supplementary material.

## Supplementary Materials

The following are available online at https://www.mdpi.com/1422-0067/22/5/2499/s1, Dataset S1: Clinical scores graded within 32 limbs on the day of stem-cell injection (before injection = Day 0) on week 3, the following day (Day 1), 3 days after injection (Day 3) and 7 days after injection (Day 7); Dataset S2: Scores and values of clinical, imaging, post-mortem and synovial fluid parameters within 32 fetlocks on weeks -1, 3, 8 and 12; Table S1: Grading systems used for the clinical evaluation of sensitivity to digital flexion tests and fetlock joint effusion; Table S2: Grading system used for ultrasound evaluation of synovial fluid effusion of the fetlock joints; Table S3: Magnetic resonance imaging parameters; Table S4: MRI grading system; Table S5: Macroscopic and microscopic grading systems.

## Abbreviations

BM	Bone-Marrow
BM-MSC	Bone-Marrow derived Mesenchymal Stem Cell
CB	Fetlocks injected with UCB-MSCs
CD	Cluster of Differentiation
CTX-II	C-terminal of type II collagen
FCS	Fetal Calf Serum
MHC	Major Histocompatibility Complex
MRI	Magnetic Resonance Imaging
MSC	Mesenchymal Stem Cell
NSAID	Non-steroidal anti-inflammatory drug
OA	Osteoarthritis
P4	Passage 4
PBS	Phosphate-Buffered Saline
PGE2	Prostaglandin E2
UCB-MSC	Umbilical Cord Blood derived Mesenchymal Stem Cell
UCB	Umbilical Cord Blood
W	Week
