# Supplementary material for: Evaluation of Allogeneic Bone-Marrow-Derived and Umbilical Cord Blood-Derived Mesenchymal Stem Cells to Prevent the Development of Osteoarthritis in An Equine Model
Source: Int J Mol Sci. 2021 Mar 2;22(5):2499. doi: 10.3390/ijms22052499 (PMC7958841; doi:10.3390/ijms22052499)
Supplement: Supplementary file 1 [file ijms-22-02499-s001.zip › Supporting information/Dataset S1.pdf]

1: left fore
2: right fore
3: left hind
4: right hind

1: Placebo
2: BM-MSCs
3: UCB-MSCs

-21: surgery
0: treatment

Horse number	Limb	Fetlock number	Treatment	Day post injection	Fetlock circumference (mean value)	Sensitivity to flexion (grade/4)	Joint effusion (grade/4)	Lameness (grade/5)
1	1	11	3	0	25,90	0	1	0
1	1	11	3	1	25,33	0	1	0
1	1	11	3	3	25,43	0	1	0
1	1	11	3	7	25,07	0	1	0
1	2	12	1	0	25,60	0	1	0
1	2	12	1	1	24,73	0	1	0
1	2	12	1	3	24,47	0	1	0
1	2	12	1	7	24,43	0	0	0
1	3	13	1	0	26,83	0	1	0
1	3	13	1	1	26,50	0	1	0
1	3	13	1	3	26,27	0	1	0
1	3	13	1	7	26,17	0	0	0
1	4	14	2	0	26,77	0	1	0
1	4	14	2	1	26,00	0	1	0
1	4	14	2	3	25,90	0	1	0
1	4	14	2	7	26,00	0	1	0
2	1	21	2	0	27,00	0	1	0
2	1	21	2	1	26,40	0	0	0
2	1	21	2	3	25,93	0	0	0
2	1	21	2	7	26,07	0	0	0
2	2	22	1	0	26,37	0	1	0
2	2	22	1	1	26,00	0	0	0
2	2	22	1	3	26,13	0	0	0
2	2	22	1	7	26,53	0	0	0
2	3	23	3	0	28,70	0	2	0
2	3	23	3	1	27,67	0	2	0
2	3	23	3	3	27,97	0	1	0
2	3	23	3	7	27,80	0	1	0
2	4	24	1	0	28,53	0	2	0
2	4	24	1	1	27,57	0	2	0
2	4	24	1	3	28,27	0	2	0
2	4	24	1	7	27,97	0	2	0
3	1	31	1	0	26,40	0	2	1
3	1	31	1	1	26,57	0	2	0
3	1	31	1	3	26,83	0	1	0
3	1	31	1	7	26,57	0	1	0
3	2	32	2	0	26,50	0	2	0
3	2	32	2	1	26,80	0	2	0
3	2	32	2	3	26,87	0	2	0
3	2	32	2	7	26,40	0	1	0
3	3	33	3	0	27,30	0	3	0
3	3	33	3	1	27,43	0	4	0
3	3	33	3	3	27,67	0	3	0
3	3	33	3	7	26,87	0	3	0
3	4	34	1	0	27,83	0	3	1
3	4	34	1	1	27,97	0	3	1
3	4	34	1	3	27,90	0	3	1
3	4	34	1	7	27,50	0	3	2
4	1	41	1	0	27,23	0	2	0
4	1	41	1	1	27,23	0	2	0
4	1	41	1	3	27,13	0	2	0
4	1	41	1	7	26,53	0	2	0
4	2	42	3	0	27,07	0	2	0
4	2	42	3	1	26,90	0	2	0
4	2	42	3	3	26,83	0	3	0
4	2	42	3	7	27,00	0	3	1
4	3	43	2	0	27,83	0	3	0
4	3	43	2	1	27,50	0	3	0
4	3	43	2	3	27,83	0	3	0
4	3	43	2	7	27,87	0	3	0

4	4	44	1	0	28,50	0	2	0
4	4	44	1	1	28,00	0	2	0
4	4	44	1	3	27,70	0	2	0
4	4	44	1	7	27,23	0	1	0
5	1	51	1	0	27,07	0	1	0
5	1	51	1	1	27,40	0	1	0
5	1	51	1	3	27,73	0	0	0
5	1	51	1	7	27,53	0	0	0
5	2	52	2	0	27,13	0	1	0
5	2	52	2	1	27,93	0	2	1
5	2	52	2	3	28,23	0	2	1
5	2	52	2	7	27,47	0	2	1
5	3	53	1	0	28,90	0	3	0
5	3	53	1	1	29,03	0	3	0
5	3	53	1	3	28,97	0	3	0
5	3	53	1	7	28,87	0	3	0
5	4	54	3	0	28,67	0	3	0
5	4	54	3	1	29,87	0	4	2
5	4	54	3	3	30,27	0	3	1
5	4	54	3	7	28,50	0	3	1
6	1	61	2	0	25,00	0	1	1
6	1	61	2	1	25,07	0	1	1
6	1	61	2	3	24,93	0	0	0
6	1	61	2	7	24,90	0	0	0
6	2	62	1	0	25,37	0	0	0
6	2	62	1	1	24,93	0	0	0
6	2	62	1	3	24,93	0	0	0
6	2	62	1	7	25,00	0	0	0
6	3	63	1	0	26,30	0	2	2
6	3	63	1	1	26,40	0	2	2
6	3	63	1	3	26,97	0	2	1
6	3	63	1	7	26,40	0	0	1
6	4	64	2	0	26,17	0	0	0
6	4	64	2	1	26,13	0	1	0
6	4	64	2	3	25,90	0	1	0
6	4	64	2	7	25,80	0	0	0
7	1	71	1	0	26,83	0	2	1
7	1	71	1	1	26,70	0	2	0
7	1	71	1	3	26,70	0	2	0
7	1	71	1	7	26,83	0	0	0
7	2	72	2	0	26,83	0	2	0
7	2	72	2	1	27,03	0	3	0
7	2	72	2	3	26,87	0	2	0
7	2	72	2	7	27,23	0	2	1
7	3	73	2	0	28,80	0	2	0
7	3	73	2	1	28,77	0	1	0
7	3	73	2	3	28,90	0	1	0
7	3	73	2	7	28,87	0	1	0
7	4	74	1	0	28,17	0	1	0
7	4	74	1	1	28,77	0	1	0
7	4	74	1	3	28,43	0	1	1
7	4	74	1	7	28,17	0	1	1
8	1	81	1	0	25,63	0	1	0
8	1	81	1	1	25,23	0	1	0
8	1	81	1	3	25,33	0	0	0
8	1	81	1	7	25,37	0	1	0
8	2	82	2	0	25,73	0	1	0
8	2	82	2	1	25,07	0	1	0
8	2	82	2	3	25,47	0	0	0
8	2	82	2	7	25,90	0	0	0
8	3	83	2	0	26,73	0	2	0
8	3	83	2	1	26,50	0	2	0
8	3	83	2	3	26,57	0	2	0
8	3	83	2	7	27,10	0	2	0
8	4	84	1	0	26,80	0	2	0
8	4	84	1	1	26,47	0	2	0
8	4	84	1	3	26,10	0	2	0

8	4	84	1	7	26,93	0	2	0
