# Supplementary material for: Evaluation of Allogeneic Bone-Marrow-Derived and Umbilical Cord Blood-Derived Mesenchymal Stem Cells to Prevent the Development of Osteoarthritis in An Equine Model
Source: Int J Mol Sci. 2021 Mar 2;22(5):2499. doi: 10.3390/ijms22052499 (PMC7958841; doi:10.3390/ijms22052499)
Supplement: Supplementary file 1 [file ijms-22-02499-s001.zip › Supporting information/Table S1.pdf]

S1 Table. **Grading systems used for the clinical evaluation of sensitivity to digital flexion tests and fetlock joint effusion**

Score		Sensitivity to digital flexion tests	Fetlock joint effusion
0	Normal	No reaction of the horse to the flexion of the fetlock joint when moderate strength is used (3kg).	Concave appearance of the proximo-palmar recess of the metacarpo(tarso)phalangeal joint. No lateral swelling when medial pressure is applied to the recess.
1	Mild	The horse reacts to the flexion of the fetlock joint (withdrawal of the limb) when moderate strength is used (3kg).	Flat appearance of the proximo-palmar recess of the metacarpo(tarso)phalangeal joint. Mild lateral swelling when medial pressure is applied to the recess.
2	Moderate	The horse reacts to the flexion of the fetlock joint (withdrawal of the limb) when light strength is used (1kg).	Convex appearance of the proximo-palmar recess of the metacarpo(tarso)phalangeal joint. Lateral swelling easily obtained when medial pressure is applied to the recess.
3	Substantial	The horse reacts to the flexion of the fetlock joint (withdrawal of the limb) before strength is used.	Convex appearance of the proximo-palmar recess of the metacarpo(tarso)phalangeal joint beyond the suspensory ligament branches (third interosseous muscle). Soft consistency of the recess on palpation.
4	Severe	Violent withdrawal of the limb when flexion is applied to the fetlock joint without strength	Convex appearance of the proximo-palmar recess of the metacarpo(tarso)phalangeal joint beyond the suspensory ligament branches (third interosseous muscle) with a hard consistency of the recess on palpation, indicating synovial pressure. Synovial distension of the dorsal recess of the joint.
