# Supplementary material for: Evaluation of Allogeneic Bone-Marrow-Derived and Umbilical Cord Blood-Derived Mesenchymal Stem Cells to Prevent the Development of Osteoarthritis in An Equine Model
Source: Int J Mol Sci. 2021 Mar 2;22(5):2499. doi: 10.3390/ijms22052499 (PMC7958841; doi:10.3390/ijms22052499)
Supplement: Supplementary file 1 [file ijms-22-02499-s001.zip › Supporting information/Table S2.pdf]

S2 Table. Grading system used for ultrasound evaluation of synovial effusion of the fetlock joints

Score		Synovial fluid effusion
0	Normal	Small amount of fluid in the proximo-palmar recess of the metacapo(tarso)phalangeal joint. Concave appearance of the skin. No motion of the fluid when the recess is pressed.
1	Mild	Small amount of fluid in the proximo-palmar recess of the metacapo(tarso)phalangeal joint. Concave to flat appearance of the skin. Motion of the fluid when the recess is pressed.
2	Moderate	Moderate amount of fluid in the proximo-palmar recess of the metacapo(tarso)phalangeal joint. Convex appearance of the skin. Motion of the fluid when the recess is pressed.
3	Substantial	Substantial amount of fluid in the proximo-palmar recess of the metacapo(tarso)phalangeal joint and small amount of fluid in the dorsal recess of the joint. Convex appearance of the skin.
4	Severe	Large amount of fluid in the proximo-palmar recess of the metacapo(tarso)phalangeal joint and substantial amount of fluid on the dorsal recess of the joint with synovial pressure. Convex appearance of the skin.
