# Supplementary material for: Evaluation of Allogeneic Bone-Marrow-Derived and Umbilical Cord Blood-Derived Mesenchymal Stem Cells to Prevent the Development of Osteoarthritis in An Equine Model
Source: Int J Mol Sci. 2021 Mar 2;22(5):2499. doi: 10.3390/ijms22052499 (PMC7958841; doi:10.3390/ijms22052499)
Supplement: Supplementary file 1 [file ijms-22-02499-s001.zip › Supporting information/Table S3.pdf]

S3 Table. Magnetic Resonance Imaging Parameters

Sequence	Acquisition type	Slice thickness/ interslice gap (mm)	TE (msec)	TR (msec)	FOV	FA	Pixel Band Width
**T1-GRE UFAST**	2D	5.0/1.0	8	52	160x160	50	49
**T1-GRE HRUFAST**	2D	3.0/1.0	8	52	180x180	50	49
**T2-FSE MI**	2D	5.0/1.0	88	1544	175x175	90	65
**STIR-FSE MI**	2D	5.0/1.0	22	2536	175x 175	90	65

TE – echo time; TR – repetition time; FA – flip angle; FOV – field of view; GRE – gradient echo; FSE – fast spin echo; STIR – short tau inversion recovery; MI – motion insensitive
