# Supplementary material for: Evaluation of Allogeneic Bone-Marrow-Derived and Umbilical Cord Blood-Derived Mesenchymal Stem Cells to Prevent the Development of Osteoarthritis in An Equine Model
Source: Int J Mol Sci. 2021 Mar 2;22(5):2499. doi: 10.3390/ijms22052499 (PMC7958841; doi:10.3390/ijms22052499)
Supplement: Supplementary file 1 [file ijms-22-02499-s001.zip › Supporting information/Table S4.pdf]

S4 Table. : Magnetic resonance imaging grading system

MRI criteria	Maximal score
Synovial fluid effusion (/3)	3
Synovial membrane thickening (/3)	3
Joint capsule oedema (/3)	3
Joint capsule thickening (/3)	3
Metacarpal/tarsal subchondral bone sclerosis (/3)	3
Proximal phalanx subchondral bone sclerosis (/3)	3
Metacarpal/tarsal oedema-like lesions (/3)	3
Proximal phalanx oedema-like lesions (/3)	3
Osteophyte formation (/3)	3
**TOTAL**	**27**
