# Supplementary material for: Evaluation of Allogeneic Bone-Marrow-Derived and Umbilical Cord Blood-Derived Mesenchymal Stem Cells to Prevent the Development of Osteoarthritis in An Equine Model
Source: Int J Mol Sci. 2021 Mar 2;22(5):2499. doi: 10.3390/ijms22052499 (PMC7958841; doi:10.3390/ijms22052499)
Supplement: Supplementary file 1 [file ijms-22-02499-s001.zip › Supporting information/Table S5.pdf]

S5 Table. : Macroscopic and microscopic grading systems

Macroscopic Score		Microscopic Score	
Wear lines	/3	Severity of the lesion	/6
Erosions	/3	Extension of the lesion	/4
Palmar/plantar osteochondral lesions	/3	Depth of the lesion	/4
**TOTAL**	**/9**	**TOTAL**	**/96**
